# Nanoelectrospray based synthesis of large, transportable membranes with integrated membrane proteins

**DOI:** 10.1038/s41598-024-76797-w

**Published:** 2024-10-24

**Authors:** Matthias Wilm

**Affiliations:** 1 Physics Institute of the University Münster, Surface Science, Münster, Germany; 2 European Molecular Biology Laboratory (EMBL), Heidelberg, Germany; 3 Max Planck Institute for Biophysics, Frankfurt, Germany; 4 Conway Institute, University College Dublin, Belfield, Dublin 4, Ireland

**Keywords:** Membrane biophysics, Membrane structure and assembly, Molecular biophysics, Nanoscale biophysics, Biomaterials - proteins, Surfaces, interfaces and thin films

## Abstract

Membrane proteins tend to be difficult to study since they need to be integrated into a lipid bilayer membrane to function properly. This study presents a method to synthesize a macroscopically large and freely transportable membrane with integrated membrane proteins which is useful for studying membrane proteins and protein complexes in isolation. The method could serve as a blueprint for the production of larger quantities of functionalised membranes for integration into technical devices similar to the MinION DNA sequencer. It is possible to self-assemble larger biological membranes on solid surfaces. However, they cannot be removed from their solid support without destroying them. In transportable form, self-assembled membranes are limited to sizes of about 17 nm in nanodiscs. Here we electrospray a series of molecular layers onto the liquid surface of a buffer solution which creates a flat, liquid environment on the surface that directs the self-assembly of the membrane. This method enables us to experimentally control the membrane composition and to succeed in producing large membranes with integrated OmpG, a transmembrane pore protein. The technique is compatible with the assembly of membrane based protein complexes. Listeriolysin O and pneumolysin efficiently assemble into non-covalent membrane pore complexes of approximately 30 units or more within the surface layer.

**Supplementary Information:**

The online version contains supplementary material available at 10.1038/s41598-024-76797-w.

## Introduction

The aim of this study is to make membranes with integrated proteins accessible for functional and structural studies. We are using a nanoelectrospray device to synthesize an environment from gas phase in which the membranes can self-assemble. The molecular composition of the membrane, its lipid and proteinaceous components, is controlled by the experimental design.

**Fig. 1:**
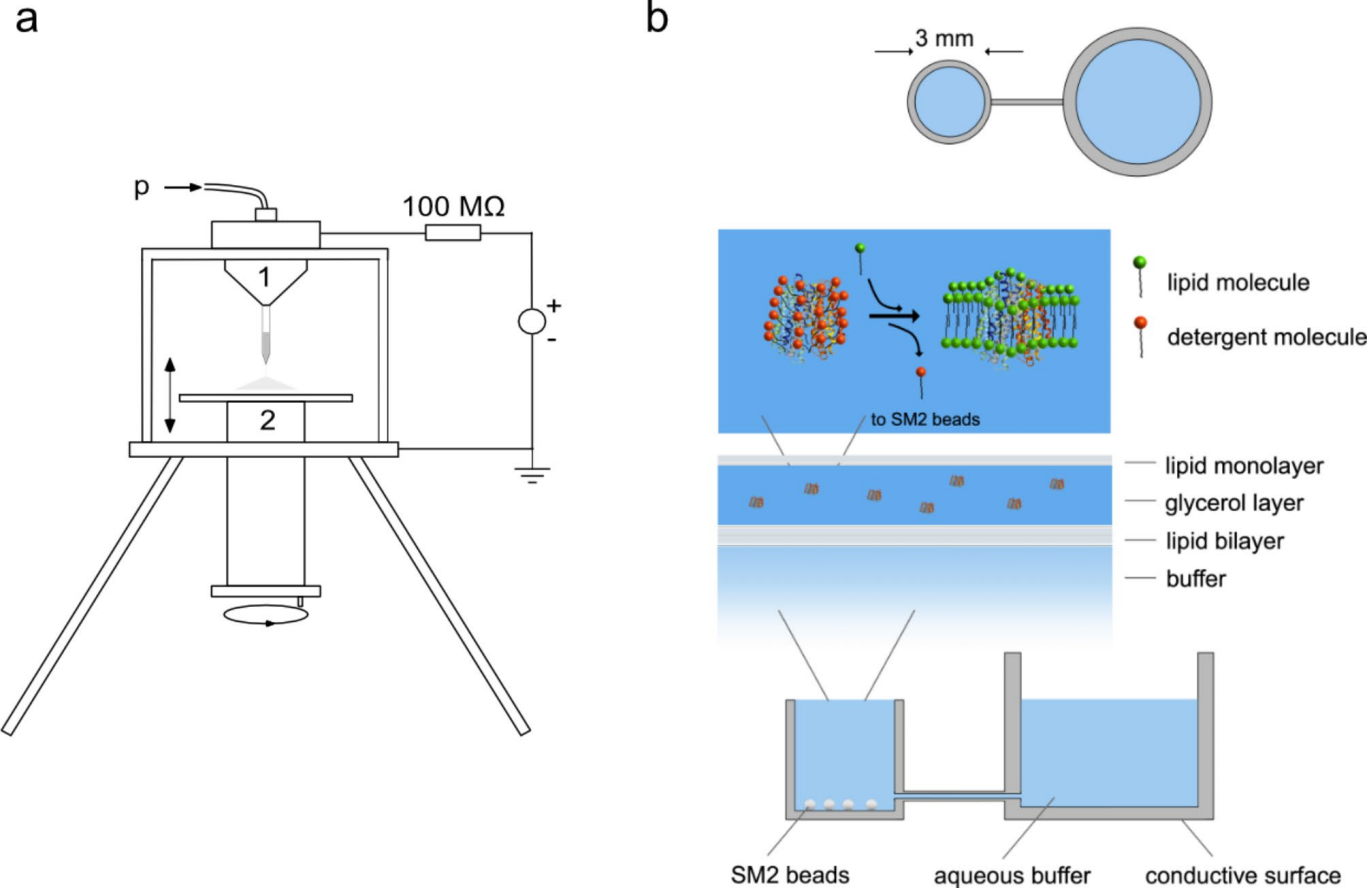
Electrospray apparatus for surface preparation. Panel (**a**) shows the electrospray apparatus for membrane preparation (2nd generation instrument): A pressurized container holds a gold-plated, drawn glass capillary filled with 1 or 2 µL solution (1). An optional low pressure supports the continuous flow of sample through the glass capillary during operation. The distance between the grounded target (2) and the needle is adjustable. It was usually 3 cm. The first generation instrument had a 250 μm steel nozzle and a manually drawn glass capillary as the emitter. Its vessel was not pressurized^7^. Panel (**b**) shows how this instrument was used to create a layered environment for the self-assembly of large membranes with membrane proteins. The spray was directed at the liquid surface in a conductive container filled with buffer solution with some SM2 beads at the bottom. First, a lipid bilayer was formed by spraying the appropriate amounts of phosphatidylcholine. A thin layer of glycerol was then added. Finally, the detergent-solubilized membrane protein was added with some lipid to form a closed membrane together with the protein. During incubation, the SM2 beads slowly remove the detergent molecules. They are replaced on the membrane proteins by lipid molecules that promote the self-assembly of a membrane in the thin glycerol layer.

We use a nanoelectrospray ion source to create a planar environment for self-assembly of large lipid bilayers containing membrane proteins. In order to maintain the proteins’ three-dimensional shape, the technique was conducted under atmospheric conditions and ambient temperatures (see Fig. 1 panel a)^1^.

In the meantime the same technique is used to prepare soluble proteins in a thin layer of water for a cryo-EM analysis^2^. In this publication the scientists observe similar characteristics for the preparation which were found here empirically to be required for membrane self-assembly - most noteworthy the hydrophilic enclosure of the proteins.

Larger membranes have thus far only been assembled on solid supports^3^. The drawback of this method is that once formed, the membranes cannot be removed from the surface without damaging their integrity. In this study, the membrane is assembled within a thin glycerol layer on a liquid buffer surface. Once assembled, the membrane can be easily moved, for instance to a transmission electron microscope for inspection.

Completely unassisted self assembly of membranes in solution is difficult to achieve, is limited in size and can easily lead to the formation of membrane stacks, roles or vesicles^4^. Transportable membranes can otherwise be assembled in the form of nanodiscs^5^. However, these nanodiscs are limited to a diameter of approximately 17 nm, as they become unstable and collapse beyond that point. Here, the self-assembly takes place in a thin layer which prevents the membrane from forming stacks, spheres, or rolls.

The nanoelectrospray method utilized in this study was established over 30 years ago. Relevant experiments have been included as they provided the base of these experiments and have not been published before.

## Results

### Nanoelectrospay as a molecular beam device

#### Nanoelectrospray based surface preparation

**Fig. 2:**
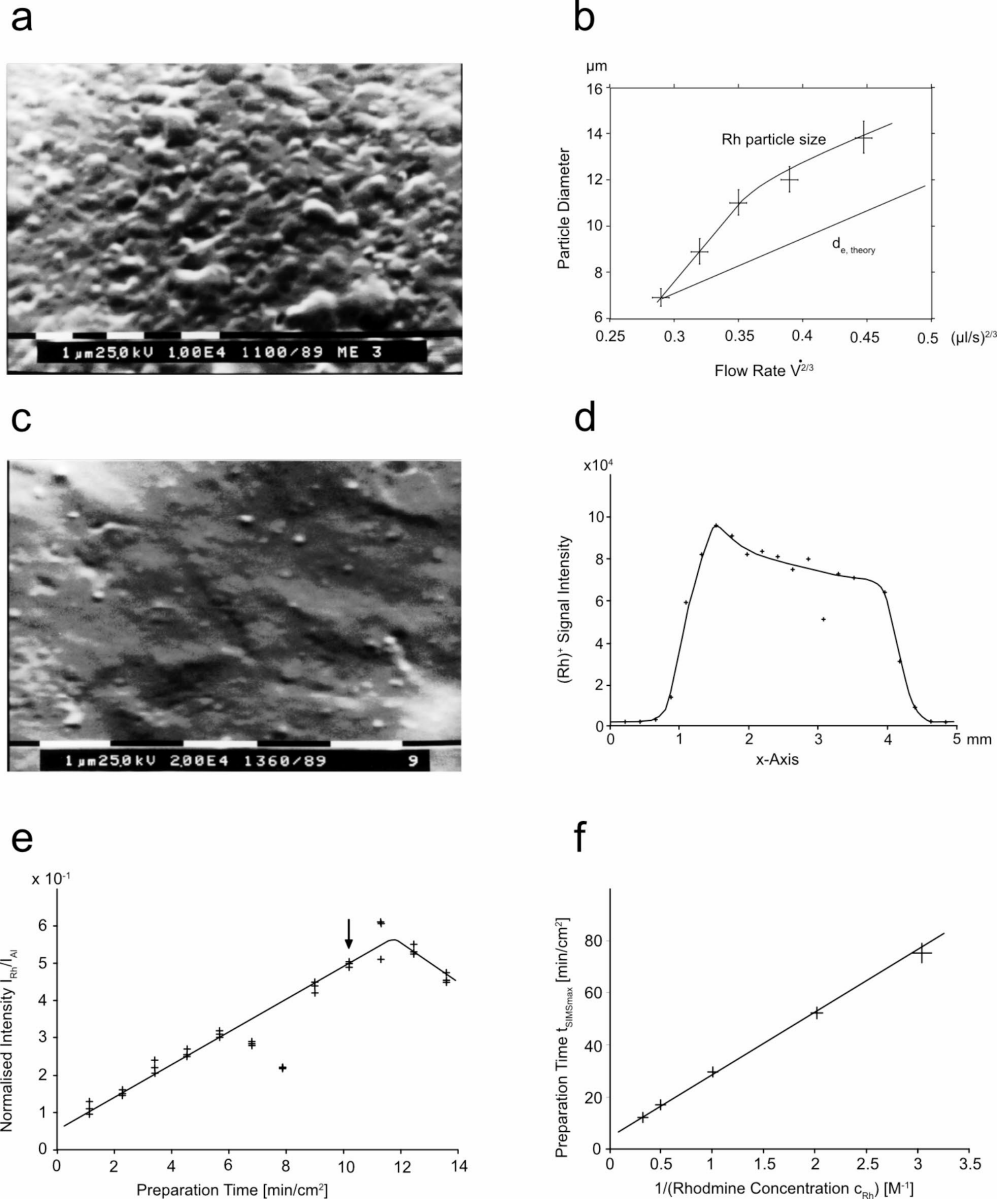
Electrospray prepared surface. Panel (**a**) shows the raster electron microscopic image of a surface prepared by electrospraying from a 250 μm nozzle. The surface layer was deposited from a 30 µM rhodamine acetone solution sprayed onto a metal target at a flow rate of 8–15 µL/min. The image shows a 10 000x magnification of the gold-coated rhodamine surface. The surface is densely covered with 0.2–1.5 μm particles. Panel (**b**) shows that the maximum observed particle size on the target depends on the flow rate in accordance with formula 1. When spraying a saturated rhodamine/acetone solution, the largest particle diameter on the target is approximately the size of the initial droplets. Panel (**b**) had been published before when describing the electrospray theory^7^. Panel (**c**) shows a layer prepared with rhodamine using a self-sustained flow rate of approximately 200 nL/min generated with a hand-pulled gold-coated glass capillary. The photo shows the surface magnified 20 000 times. While rhodamine covered the entire surface (see panel **d**), no specific particle sizes could be determined. Panel (**d**) shows the static SIMS signal of the same target as in panel (**c**), but before it was coated with gold. The spatially resolved static SIMS showed that the rhodamine uniformly covered a 4 mm wide area. Panel (**e**) shows how the static SIMS Rh^+^ signal as a function of the amount of rhodamine sprayed onto the surface. The liquid sprayed was a 30 µM rhodamine-acetone solution. For each target, three different measurements at different locations on the target contribute to the analysis. The SIMS signal intensity increases as the surface coverage increases, passes through a maximum, and then decreases. This characteristic is similar to clean surfaces successively covered by molecular beams in a vacuum. During the preparation of targets 6 and 7 the electrospray became temporarily unstable. Target 9 was used for the panels (** c**) and (**d**). Panel (**f**) shows the relationship between the time taken to reach the maximum SIMS response signal t_SIMSmax_ and the concentration of rhodamine in the sprayed solution. This time is indirectly proportional to the rhodamine concentration.

Electrospray is routinely used to prepare thin films^6^ even though molecular-level design is not achieved. We used a home-built electrospray device to distribute a dissolved component uniformly over a metal target (Fig. 1, panel a). Figure 2 panel a,b show the surface prepared by spraying a rhodamine solution onto a flat target with a conventional instrument built in the late 1980s of the 20th century at the Physics Department of the University of Münster, Germany^7^. The electrospray was operated at flow rates of 10–20 µL/min. The purpose was to distribute an analyte uniformly over a surface for subsequent analysis by a static secondary ion mass spectrometer (SIMS). Static SIMS is a sub-monolayer sensitive surface analysis technique. Such an electrospray preparation was state of the art at the time^6^, but far from monolayer characteristics. Subsequent theoretical and experimental studies of the electrospray process itself showed a way to improve the homogeneity of the preparation^7^. A stable liquid Taylor cone ejects droplets exclusively from its tip. The radius at the tip of the Taylor cone for droplet emission depends on the flow rate. The central equation describing the radius of the emission region from the tip of a Taylor cone is^7^:

1 $$r_{e} = \left( {\frac{\rho }{{4\pi ^{2} \gamma \,\tan \left( {\frac{\pi }{2} - \vartheta } \right) \cdot \left[ {\left( {\frac{{U_{a} }}{{U_{T} }}} \right)^{2} - 1} \right]}}} \right)^{{1/3}} \cdot \dot{V}^{{2/3}} .$$

*r*_e_: emission radius of droplets at the tip of the Taylor cone, *ρ*: density of the solution, *γ*: surface tension, *ϑ*: opening angle of the Taylor cone (49.3° in the static case ), *U*_a_: applied and *U*_T_ Taylor cone threshold voltage, $\dot{V}$flow rate.

The central equation linking the radius r_e_ of the droplet emission region at the tip of a Taylor cone with the flow rate V’. The lower the flow rate the smaller are the initially produced droplets.

Reducing the flow rate reduces the size of the initially produced droplets (Fig. 2, panel b). The equation suggests that when spraying an aqueous solution of 1 picomoles/µL at a flow rate of 20 nL/min, droplets with the size of the emission radius would contain, on average, only one analyte molecule.

Acetone has a high vapor pressure. When sprayed its droplets start to evaporate in flight and break up into smaller second generation droplets before they dry down and the non volatile rhodamine residuals reach the surface^8^. The droplet emission radius is only a description for the largest droplet generated by the spray, not the most common one. By replacing the metallic nozzle with a gold-coated, pulled glass capillary, the acetone flow rate was reduced to about 200 nL/min. This was the first - later called - nanoelectrospray instrument. 200 nL/min was the lowest flow rate achievable at the time with the first generation instrument. With this emitter, the metallic aluminum surface did not visibly change when the rhodamine solution was sprayed - even under prolonged preparation of 1 h or more. The obtained surfaces were investigated with a raster-electron microscope and a static SIMS mass spectrometer (Fig. 2, panel c,d). The raster electron microscopic picture showed no discernible particles even though rhodamine covered the surface of the target.

The following experiments were designed to determine whether the nanoelectrospray produced surfaces that behaved like monolayers under static SIMS examination. Such a preparation would allow the hypothesis that the final droplets were so small that they contained on average only one molecule of rhodamine. The experimental results obtained at this stage were not completely conclusive, but they provided enough evidence to continue this line of experimentation.

Figure 2, panel e shows the slow increase of the static SIMS signal with preparation time and a decrease when the rhodamine coverage becomes too thick. This behavior is similar to monolayer coverage on clean surfaces in vacuum by molecular beam deposition^9^. The question was whether the surface is covered by an increasing number of particles, as in panel a, or whether the coverage is monolayer-like. To answer this question, a series of experiments were performed to determine the time required to reach the maximum SIMS response signal t_SIMSmax_ as a function of rhodamine concentration. When small rhodamine particles cover the target, t_SIMSmax_ depends on their cross section and thus varies with (c_Rh_)^-2/3^. However, if the coverage is monolayer-like, the static SIMS signal will respond to the total amount of rhodamine sprayed on the surface and will depend on (c_Rh_)^-1^. Figure 2 panel f shows how the preparation time to reach the maximum SIMS signal t is indirectly proportional to the rhodamine concentration. The linearity indicates that the SIMS signal characteristic changes in proportion to the total amount of rhodamine sprayed onto the target. This result indicates that the surfaces generated by the nanoelectrospray preparation behave like molecular monolayers under static SIMS examination.

#### Molecular beam hypothesis

The surface preparation experiments concluded that when an acetone solution is electrosprayed from a stable Taylor cone using a metal-coated drawn glass capillary at a flow rate of the order of 200 nL/min, the compartmentalization of individual analyte molecules into separate droplets is complete or close to complete. Two hypotheses emerged from these experiments.

First, if such a device is pointed at a vacuum system, the volatile solvent would evaporate, releasing the non-volatile molecules into the vacuum. Thus, a nanoelectrospray could be an ion source for large molecules of virtually unlimited mass - which is what electrospray ion sources are today. And second, it should be possible to use a nanoelectrospray to synthesize molecularly designed layers of large organic compounds^10^.

Prof. Fenn et al. had published the first mass spectra of protein ions generated with a conventional electrospray ion source in 1988^11,12^. They had no experimental evidence suggesting that the electrospray can produce droplets so small that they contain only one protein molecule. To explain the generation of these ions from larger droplets they proposed the ion evaporation mechanism^12,13^.

**Fig. 3:**
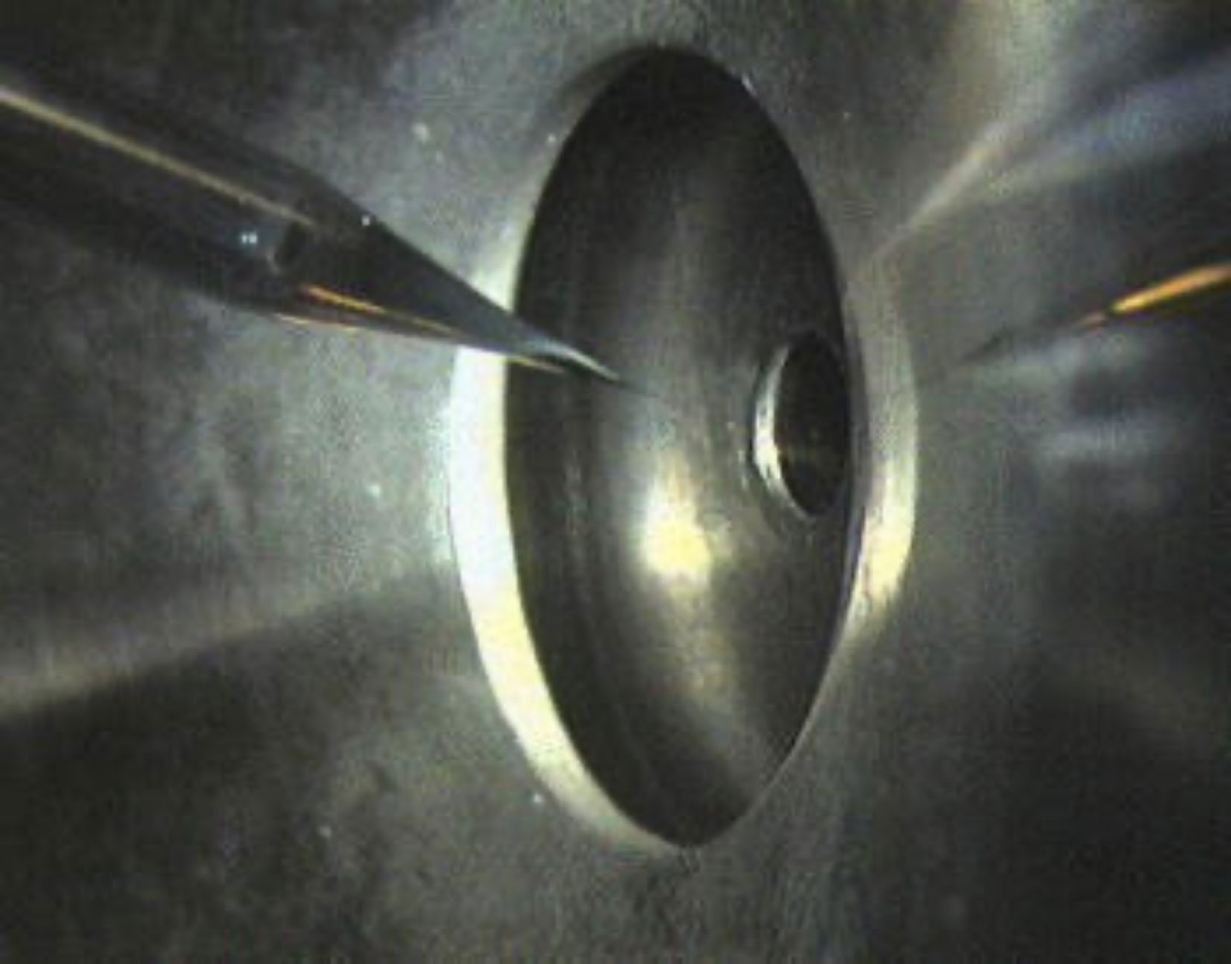
Nanoelectrospray ion source on a mass spectrometer. The nanoelectrospray ion source mounted on a triple quadrupole mass spectrometer (API III, Sciex). The image shows the tip of the emitter mounted in the same pressurized holder as in Fig. 1. The drawn glass needle is placed in the center, directly in front of the orifice to the vacuum system of the mass spectrometer. A flight path of about 1 cm under atmospheric conditions is sufficient to allow complete desolvation of large ions from aqueous solutions. No protein clusters were observed in the mass spectra.

The first project, the implementation of the nanoelectrospray as an ion source at a mass spectrometer, was realized in 1992 at the European Molecular Biology Laboratory (EMBL), Heidelberg, Germany^14^. The direct coupling of the nanoelectrospray ion source to the vacuum system of a mass spectrometer, as shown in Fig. 3, demonstrated that the compartmentalization of analyte molecules is indeed complete. Under standard operational conditions no clustering of proteins was observed. The nanoelectrospray ion source is a very bright ion source, approximately 100 times more efficient than any other electrospray ion source at the time. The instrument provided the technical basis for the development of low-level protein identification by mass spectrometry. Over the years, it has been used to analyze hundreds of peptide and protein samples.

### Self assembly of large protein containing membranes

Can a nanoelectrospray ion source be used to synthesize membranes with incorporated membrane proteins from gas phase? Because purified membrane proteins are only stable when solubilized with detergent molecules, it is not possible to synthesize such membranes directly^15,16^. The question was whether nanoelectrosprayed layers could provide an environment in which membranes can self-assemble. The experimental basis for this was the ability of the nanoelectrospray to generate monomolecular films. The question to be addressed was: What are the requirements for self-assembly of protein-filled lipid bilayers when synthesized in a layered fashion? The course of the experiments suggested the answer: self assembly starts with a lipid monolayer as template and hydrostatic stabilization of the membrane proteins on both sides of the lipid bilayer. Although a lipid bilayer can form on the surface of a liquid, protein incorporation will only occur if a hydrostatic layer thick enough to enclose the proteins surrounds the lipid layer. This experimental finding is consistent with the observation that membrane proteins in a supported bilayer can denature if the distance between the membrane and the underlying support is too small^3^.

Recently, Yang et al. have confirmed this approach of using nanoelectrospray or low-flow electrospray to design flat films at the molecular level. They were able to prepare a thin aqueous film containing soluble proteins for cryo-EM studies^2^. Part of the prepared EM grid contained a layer thin enough to reconstruct the protein structure from the images. The researchers made the same observation as reported here, that in protein structure-preserving monolayers, the proteins are surrounded by a hydrophilic shell.

The first experimental result was the observation that lipid mono- and bilayers can assemble on a liquid surface when the lipids are sprayed onto it. The substrate chosen for layer formation was the liquid meniscus of a small round container with a diameter of 3 mm, the size of a standard electron microscope grid. This corresponds to a surface area of 7.07 mm^2^. With 66 picomoles phosphatidylcholine evenly distributed over the surface the viscosity visibly changed and folds appeared in the surface after overnight incubation (see Supplementary Fig. 1). The viscosity change and folding of the surface did not occur when the layer contained only 33 picomoles. This was taken as experimental evidence that 66 of picomoles phosphatidylcholine was sufficient to form a lipid bilayer. This conclusion is consistent with the calculated surface occupancy of the phosphatidylcholine molecules. 33 picomoles phosphatidylcholine are 1.9876 × 10^13^ molecules. They are evenly distributed over the circular meniscus with a diameter of 3 mm. These are 7.069 × 10^14^ Å^2^. Hence the average space occupied by each phosphatidylcholine molecule is 36 Å^2^. 36 Å^2^ lies between the surface occupancy of 60 Å^2^ − 70 Å^2^ measured for phosphatidylcholine molecules in vesicles^17^ and approximately 20 Å^2^ for a saturated fatty acid molecule in a regular Langmuir-Blodgett film^18^. Considering the linear structure of saturated fatty acids and their dense packing in a regular array, 20 Å^2^ is certainly too small for the surface occupancy of a phosphatidylcholine molecule in a closed, flat phosphatidylcholine film. With the globular shape of vesicles 60 Å^2^ for a single phosphatidylcholine molecule is more than this molecule would occupy in a flat layer. Hence, 36 Å^2^ as calculated from the experimentally found conditions for the preparation of a monolayer is within in the expected range.

The behavior of Escherichia coli outer membrane protein G (OmpG) on surfaces generated by spraying 66 picomoles and 33 picomoles underscores their different nature. OmpG is a relatively robust 35 kDa monomeric channel protein. It is a membrane-spanning protein that denatures in an aqueous environment lacking detergent to cover its hydrophobic regions. Its crystal structure shows that its pore has an inner diameter of 2 –2.5 nm^15,16^. It was observed that OmpG sprayed onto a surface prepared with 66 picomoles phosphatidylcholine denatures upon removal of the detergent. When sprayed onto a surface with only 33 picomoles phosphatidylcholine, it remains intact.

**Fig. 4:**
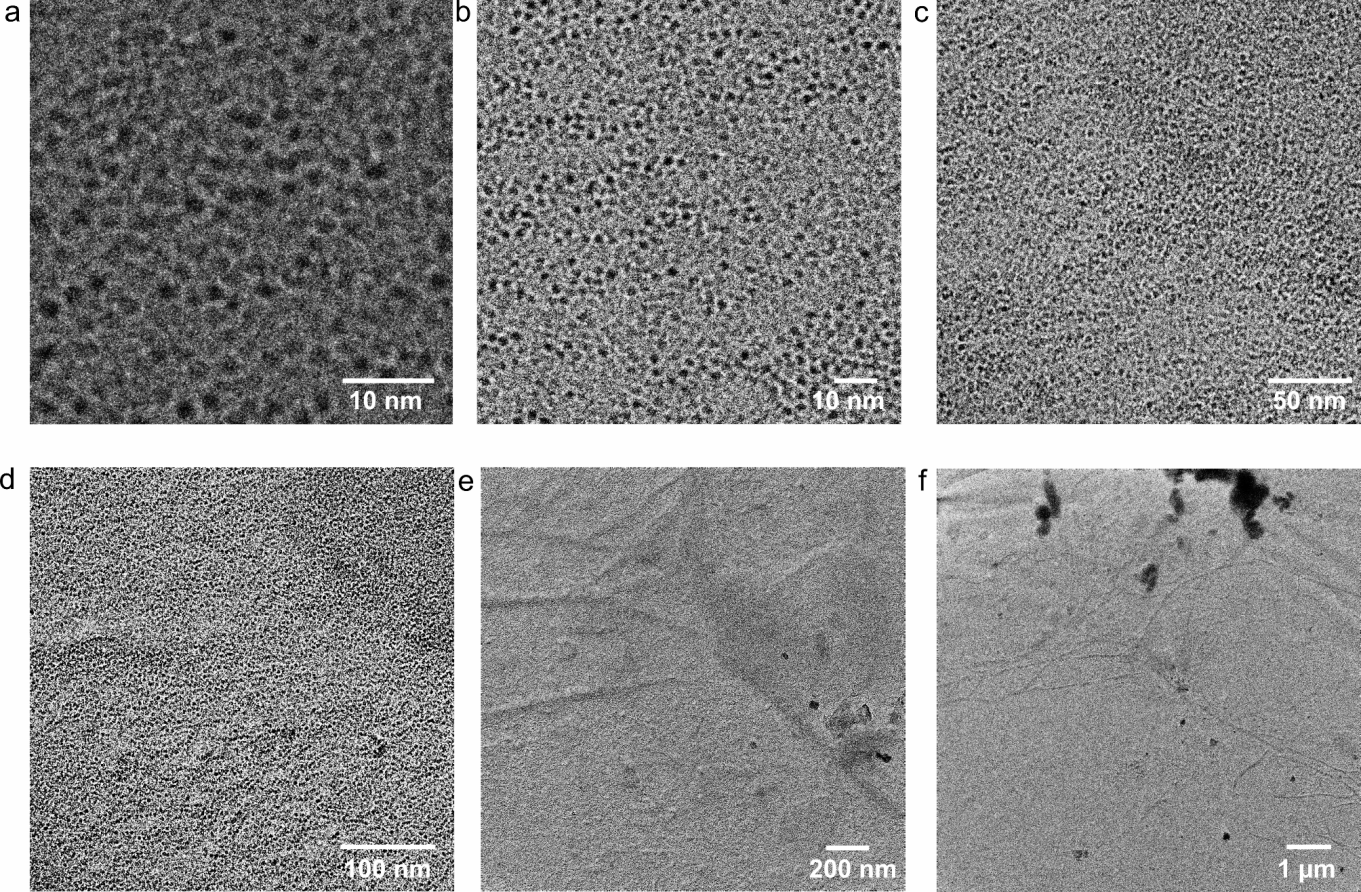
Large OmpG containing membrane prepared by nanoelectrospray in a layered fashion. The images show a transmission electron microscopy investigation of a nanoelectrosprayed OmpG-containing membrane shadowed by a 1.5 nm thick platinum layer at an angle of 15°. The images show central zoom-outs from the highest resolution image in panel a to capture the self-similarity of the homogeneous layer. Panel (**a**) shows the membrane at the highest magnification and resolution. A monolayer of OmpG-filled membrane covers the entire field of view. The image in panel (**b**) has a magnification one step lower. The OmpG pores are still well resolved and occupy the entire field of view. In panel (**c**), the zoom-out process is continued. The OmpG pores are no longer well resolved, but the membrane is still homogeneous. Panel (**d**) shows a larger area. OmpG pores appear as a granular background. In panels (**e**) and (**f**), the field of view is finally so large that no individual pores can be resolved. However, the membrane is still homogeneous with the exception of some dust particles at the top of the image in panel (**f**). The observed size and shape of the structures correspond very well to the space filling model derived from crystallization data of OmpG [16] (see Supplementary Movie 1).

The preparation protocol to generate large membrane layers in a reproducible and robust manner (Fig. 4) has several steps. The first step consists of spraying 66 picomoles of bipolar lipid onto a buffer containing SM-2 biobeads. The vessel was incubated overnight at room temperature. This creates a lipid bilayer that retains subsequent molecules at the surface. A thin layer of glycerol lays the foundation for membrane assembly in a hydrophilic environment by spraying a 1:2 glycerol/ethanol solution. The equivalent of a lipid monolayer is added (33 picomoles phosphatidylcholine) followed by the detergent solution of OmpG and enough lipid to form a closed bilayer (20 picomoles phosphatidylcholine) (see Fig. 1, panel b). A final glycerol layer completes the hydrophilic environment. Empirically, it is necessary to add this final layer of glycerol to promote membrane formation. Mechanistically, it is not entirely clear why this is so. The additional glycerol may be necessary to have a sufficiently thick glycerol layer to encapsulate the forming membrane. Alternatively, the glycerol may convert the lipid monolayer on the surface into vesicles that can then contribute to membrane formation. The OmpG membrane formation was highly reproducible. Once this protocol was established it worked six times in six experiments.

The SM-2 beads in the buffer extracted the detergent from the surface during a six-days incubation period. After transfer to an EM grid and washing with distilled water, the grid was exposed to a platinum beam in vacuo. Figure 4 shows a series of images generated from such EM grids. A single, extended, intact OmpG-containing layer has formed. No denatured protein was visible. OmpG denatures in an aqueous environment unless stabilized by detergents or lipids. The presence of the observed structures depends on the addition of OmpG to the surface. The structures were compared with crystallization data of OmpG. In the membrane images, the OmpG pores have an average outer diameter of 2.9 nm with a standard deviation of 0.2 and an average inner pore dimension of 1.2 nm with a standard deviation of 0.1. This compares to 2.1 ± 0.1 nm for the outer diameter and approximately 1.3 nm for the inner pore from crystallization data^16^. Considering that the EM images are at the limit of the instrument resolution, this is an acceptable agreement. Movie 1 (Supplementary Material) visualizes this agreement between the space-filling model derived from the crystallization data and the images presented here. When the membrane was successfully assembled it usually covered the entire EM grid and was as such visible where the carbon layer was intact and no dust particles blocked the view. This is similar to the successful self-assembly of membranes on solid support surfaces, where surface coverage reaches 84–94% under favourable conditions^19^. This coverage tends to be dependent on lipid concentrations and surface quality, as self-assembly occurs from a volume phase. Electrospray based self assembly on the other hand is a surface effect and is therefore dependent on the total amount of lipid and the size of the available surface area.

### Protein complex formation in nanoelectrospray generated layers

The assembly of a large protein-containing membrane occurs in a thin layer of glycerol, which is probably too thin to promote the formation of membrane stacks or tubes. The question is whether this highly artificial environment allows the assembly of membrane-based protein complexes. Although this has to be decided on a case-by-case basis, we have tested this using the proteins listeriolysin O and pneumolysin. Listeriolysin O and pneumolysin are both members of a group of pore-forming toxins. They are soluble 53 kDa and 60 kDa proteins, respectively. Up to 50 monomers form a non-covalent ring-shaped complex on membranes. The rings insert themselves and create large pores of about 30 nm in diameter^20,21^. Listeriolysin O assembles best at a slightly acidic pH of 5.5. Both proteins require phospholipids in the membrane for docking and complex formation.

#### Listeriolysin O assembly on a lipid bilayer

**Fig. 5:**
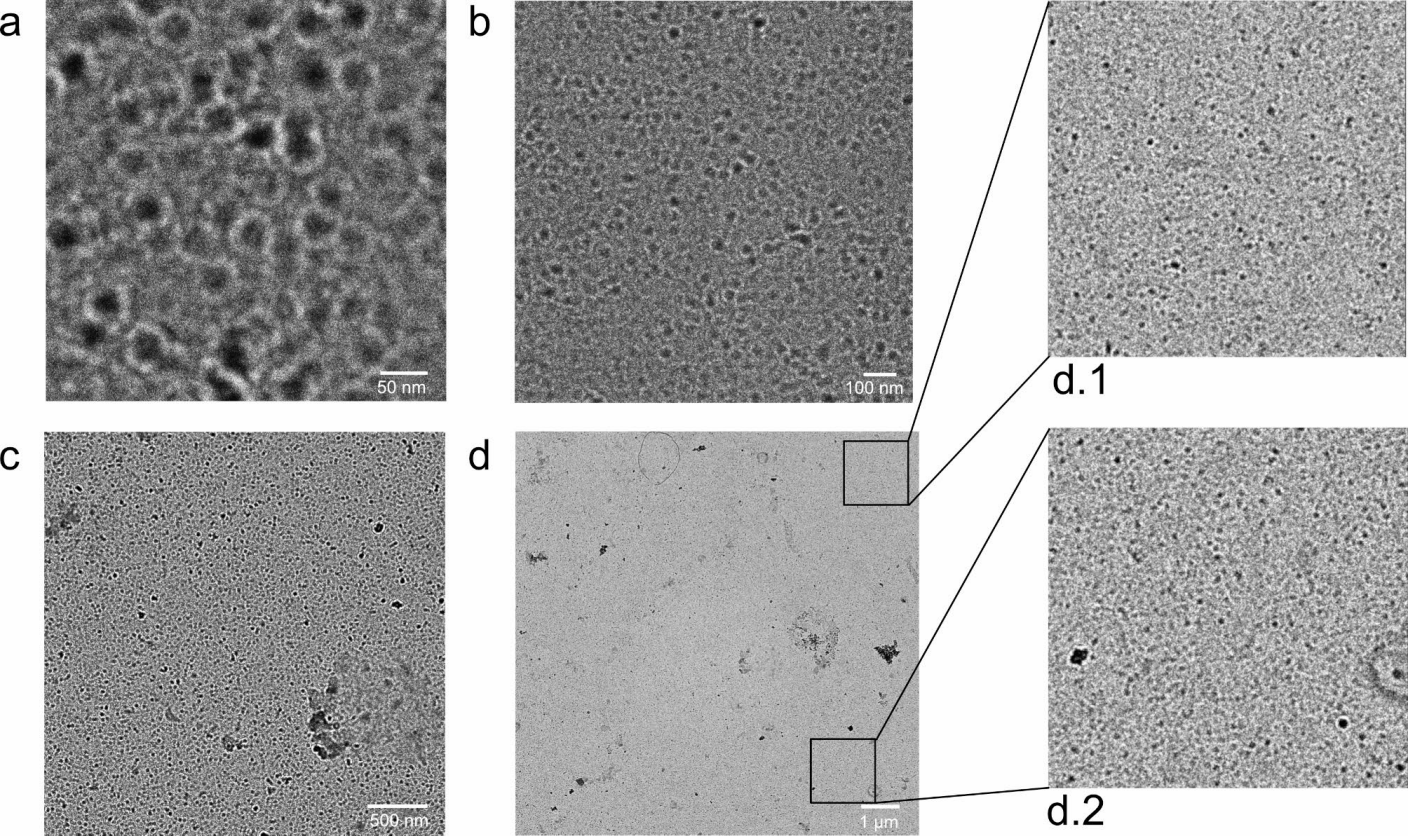
Listeriolysin O assembly on a lipid bilayer. Transmission electron microscope image of listeriolysin O sprayed onto a lipid bilayer and covered with glycerol: negative staining with 1% uranyl acetate solution enhances the contrast. Panel (**a**) shows the membrane at the highest magnification and resolution. Pores made of listeriolysin O monomers cover the entire field of view. The image in panel (**b**) has half the magnification. The large pores still occupy the entire field of view. Panel (**c**) shows an even larger area. The pores are no longer well resolved, but the membrane is still homogeneous, except for some surface contamination in the lower right corner. Panel (**d**) shows such a large area that no pores can be resolved. However, blow-ups from the lower and upper corners in panels (**d.1**) and (**d.2**) show that the membrane is still full of pores and homogeneous. Pores assembled on supported lipid bilayers have a comparable density and shape^32^.

The buffer chosen for the preparation of listeriolysin O layers allows the assembly of its complexes on membranes^22^. However, in these experiments, the local environment for the protein is a thin layer of glycerol, not the buffer itself. In thin layers, ion concentrations can be very different from bulk solution. In an experiment to test complex formation, the nanoelectrospray first generated a lipid bilayer film, then a film containing listeriolysin O, followed by a glycerol layer to complete the hydrophilic environment. Figure 5 shows the result. The overall appearance is consistent with published listeriolysin O complexes on membranes^22^. The observed pores have an average inner diameter of 22.5 nm with a standard deviation of 2 nm and an average outer diameter of 47 nm with a standard deviation of 4 nm. This compares well with the geometry of listeriolysin complexes observed by atomic force microscopy on planar lipid layers with an outer diameter of 45–50 nm and an inner diameter of 30 nm, taking into account the lower resolution of the microscope^22^. The assembly of the complex is highly efficient. The electron dense centers of many of the completed rings are probably due to residues of glycerol and salt from the buffer solution that were not successfully removed by the washing procedure. These complex formations were a reproducible result. From three experiments two were successful.

#### Listeriolysin O and pneumolysin assembly on a lipid monolayer

**Fig. 6:**
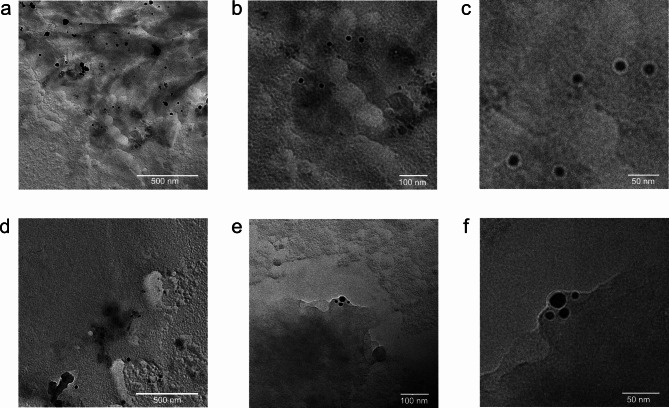
Listeriolysin O and pneumolysin on a lipid monolayer. Pneumolysin and listeriolysin O sprayed onto a lipid monolayer: panels (**a**–**c**) show a pneumolysin preparation at increasing resolutions, panels (**d**–**f**) the same for a listeriolysin O preparation. The samples were shadowed by a 1.5 nm thick platinum layer at an angle of 15°. Panels (**a**) and (**d**) show in overview that the surface is much more heterogeneous than in a successful preparation (see Fig. 5). Panels (**b**) and (**e**) show that some, but very few, pore-like structures can be seen and panels (**c**) and (**f**) show these pores in higher resolution. Considering their overall appearance and size, these could be pneumolysin and listeriolysin pores, but their sporadic appearance does not qualify this experiment as a successful preparation. This failure is in agreement with the pore-formation model for pneumolysin and listeriolysin O. They require a lipid bilayer containing phospholipids for assembly^20,21^.

The physiological mechanism of listeriolysin O and pneumolysin is to form a ring-shaped complex on a bilayer membrane. The ring inserts itself and forms a pore^22,23^. What would happen if the protein were prepared in the same way as an intramembrane protein, by spraying it onto a lipid monolayer ? Fig. 6 shows the result.

Some ring-like structures of the expected size are visible, but the efficiency is dramatically reduced compared to the preparation on a lipid bilayer. This is consistent with the mechanism of formation of listeriolysin and pneumolysin pores. In analogy to the experiments with OmpG, which denatures when sprayed onto a lipid bilayer upon detergent removal, the experiment shows that nanoelectrospray is indeed capable of preparing lipid mono- and bilayer structures that behave radically different when interacting with membrane proteins or protein complexes.

#### Direct observation of protein complex formation

All observations reported so far were systematic results, repeated several times. Here, we present a sporadic observation that supports the interpretation that the electron dense centers of the ring-like structures are glycerol-filled pores and highlights the potential of nanoelectrospray-based preparation methods: the direct observation of the pore formation process of pneumolysine, from monomer to finished pore.

**Fig. 7:**
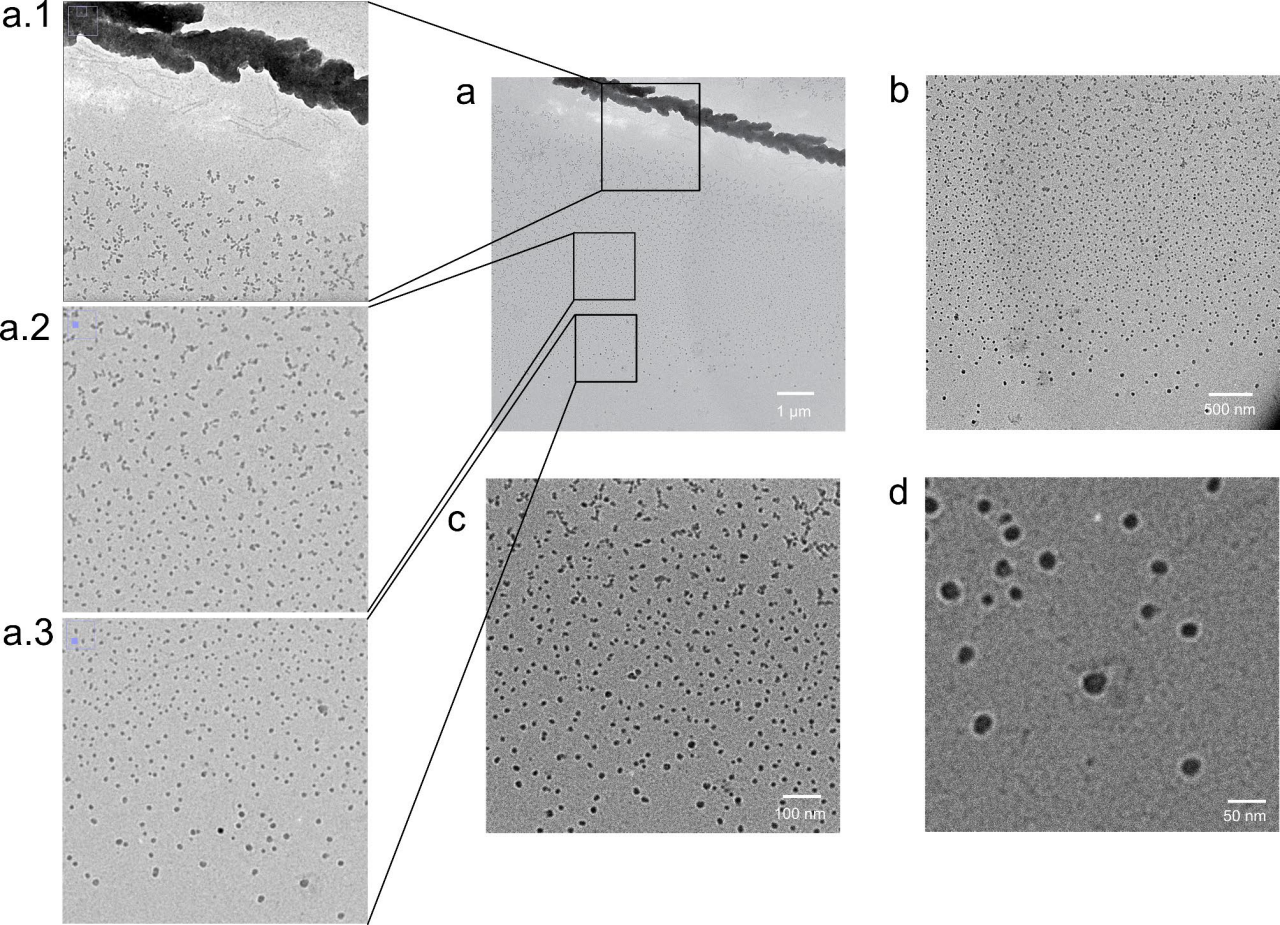
Direct observation of pneumolysin complex formation. Pneumolysin preparation on a lipid monolayer: Pneumolysin was sprayed onto a lipid monolayer and covered with glycerol. After five days of incubation, the layer was transferred to a carbon-coated grid, washed with buffer, negatively stained with 1% uranyl acetate, and washed with water. Under these conditions only very few pneumolysin pores should have formed (see Fig. 6). However, in this preparation some salt cluster from the pneumolysin buffer formed on the supporting membrane because the flow rate of the electrospray was too high (see Fig. 2). During incubation some of the pneumolysin dissolves into the glycerin layer and assembles slowly to fully formed pneumolysin pores on the membrane. The size and the shape of the forming pores correspond to the expected structures^20,21^. Panel (**a**) shows an overview image at the lowest resolution. The buffer salt crystals containing pneumolysin can be seen at the top. The blow-ups in panels (**a.1**–**a.3**) show how monomers slowly assemble into complete pores with increasing diffusion distance from the salt crystals. No structures are visible in the immediate vicinity of the salt crystal because the pneumolysin monomers are too small to be resolved by electron microscopy. Panels (**b**–**d**) show parts of the field of view of panel (**a**) with increasing resolution to better visualise the assembly process and the final pores. The electron dense centers of the pores are most likely caused by glycerol that is not completely washed out. A stepwise pore forming process is what computer simulations propose for related bacterial pore forming toxins like listeriolysin O^25^.

The images in Fig. 7 are from a preparation of pneumolysin on lipid monolayers. Initially, there should have been very few pores (see Fig. 6). However, in this case the spray was not operated at the desired low flow rate. Larger droplets formed that landed on the surface membrane and the water evaporated, leaving crystalline salt residues on the surface. Nanoelectrospray ion sources on mass spectrometers operate with volatile buffers, but for these experiments a more conservative approach was taken and the original solution in which the proteins were kept was not altered. After the preparation was complete, enough lipid was sprayed to form bilayers on the surface. The images in Fig. 7 suggest that during the incubation period, pneumolysin monomers slowly dissolve from the crystalline residue and diffuse into the glycerol layer. As they encounter a lipid bilayer, they begin to assemble and form pores. The fully formed pneumolysin pores have an average inner diameter of 19 nm with a standard deviation of 2 nm and an average outer diameter of about 47 nm with a standard deviation of 4 nm. This corresponds within the resolution limits of the electron microscope to the cryo-EM reconstructed structure of pneumolysin pores with an inner diameter of 25–26 nm and an outer diameter of 40 nm^24^. The deviation from circularity is minimal. The average difference between the horizontal and vertical pore diameter is 2% with a standard deviation of 2. This step-wise pore forming process corresponds to the pore forming mechanism proposed by computer simulations of related proteins like listeriolysin O^25^. The whole process is visible and suggests that it is slow enough to be extended over the diffusion range. Glycerol has a much higher viscosity than water. And diffusion in thin layers is slower than in bulk solution. All this suggests that the formation of protein complexes in thin layers of glycerol may be directly observable, especially if the temperature of the layer is controlled.

Pore formation in a supported bilayer has been directly observed by atomic force microscopy for the membrane attack complex (MAC)^26^. The pore is completed in approximately 100 s.

## Discussion

The second of the two original hypotheses, that nanoelectrospray should allow the composition of surfaces at the molecular level, seems to hold. The fact that it was possible to self-assemble a large OmpG-containing membrane with a lipid monolayer as base, but not with a lipid bilayer, confirms this hypothesis. The same is true for the formation of listeriolysin O and pneumolysin pores. Only when sprayed on a lipid bilayer does pore formation proceed effectively. These experiments describe a way to generate large, protein-containing membranes that are freely transportable. The vision behind these experiments is to make bio-similar membranes available as materials for technical devices. This will combine solid-state electronics with the unique properties of specific biological membrane proteins to power bioelectronics. One such device is the MinION nanopore DNA sequencer, which uses a membrane pore protein and a helicase at its core^27^. So far, the experiments have not aimed at anisotropic orientation of the proteins. However, it is likely that the proteins will often be oriented when the membrane assembly process proceeds in a static electric field. Further experiments are needed to show that membranes can be synthesized with other membrane proteins and that the proteins can be incorporated anisotropically when the membrane is assembled in an external electric field.

These experiments don’t show that such membranes have the protein-specific biochemical activity. But I think they lay the foundation for further experiments that lead to bio-similar materials that are useful in technical devices like cell-free bioreactors and protein based sensors.

## Methods

### Protein expression & purification

Yildiz et al. describe the expression and purification of the outer membrane porin G (OmpG)^15^. Briefly, E. coli expresses the amino acids 22–301 plus an N-terminal methionine of the OmpG gene having cloning it into a pET26B plasmid vector. After breaking the cells, solubilizing them in 8 M urea, 1% Triton-X 100 and 25 mM Tris-HCl, unfolded OmpG is separated from impurities via an ion exchange column separation. The protein refolds in a 1% (w/v) n-octyl-β-D-glucopyranoside (OG), 3 M urea solution. Afterward, a final ion-exchange separation process eliminates unfolded or partially folded protein from the solution. By performing ultrafiltration or dialysis with membranes having a 12 kDa weight cutoff against a buffer with 20% PEG-35,000, the protein concentration increases to approximately 50 mg/mL. A solution comprised of 10 mM Tris, 1% octylglucoside, and 20 picomoles/µL phosphatidylcholine, with a concentration of 32 picomoles/µL OmpG, acts as the base for preparing OmpG layers. To shield the protein from the high charges of the electrospray droplets, an extra 25% glycerol is added to the final spraying solution.

Listeriolysin O (LLO) was obtained from two sources. The expression and purification procedure of LLO was described in^20^. Briefly, for E. coli BL21 (DE3) expression, the gene segment encoding residues 25–529 of LLO sequence, without N-terminal secretion signal, was cloned into the plasmid pET15b, along with an N-terminal His6 tag. Once the cellular density A600 reached 1.2, the temperature was reduced from 37 °C to 30 °C to curtail cell proliferation. Introducing 1 mM isopropyl β- D-1-thiogalactopyranoside inhibited the plasmid’s Lac-repressor, initiating protein expression. After 4 h, the cells were harvested, suspended in 50 mM Tris pH 7.7, 150 mM NaCl, and disrupted using a microfluidizer (M-110 L, Microfluidics Corp., MA). After centrifugation at 12 000 g for 1 h, the supernatant was applied to an Ni-column to extract the His6-tagged protein. The protein was cleaved from the column with thrombin, concentrated on a 30 kDa cut-off membrane, and purified on a gel filtration column (Superdex200) using 25 mM Tris (pH 7.7), 150 mM NaCl as running buffer.

Alternatively, recombinant LLO from Abcam (Cambridge, UK) was used (residues 60–529, product code ab83345).

A similar method was used to obtain pneumolysin (PLY)^23^. An Escherichia coli colony expressed the His6 tagged version of the protein by cloning it into the plasmid pET15b. The cell culture was grown overnight at 37 °C. Transfer to selective medium containing 50 µg/ml ampicillin ensured that only plasmid-containing cells survived. When an A600 between 1 and 1.5 was reached, the temperature was lowered to 30 °C to reduce cell proliferation. The addition of 0.5 mM isopropyl-β-D-1-thiogalactopyranoside induced protein expression. After overnight expression, cells were harvested, suspended in lysis buffer and disrupted using a microfluidizer. The cells were then centrifuged at 185 000 g for 1 h and the supernatant was applied to a HisTrap FF column. Using a lysis buffer containing 500 mM imidazole to displace the histidine tag from its Ni + binding sites, the protein was eluted from the column. The protein fractions were diluted with a 50 mM Tris pH 7.0, 5 mM β-mercaptoethanol solution. Overnight incubation with 100 units of thrombin at 4 °C removed the His tag. Passing the protein solution through a HiTrap Q FF using a salt gradient of 0 to 1 M NaCl in a 50 mM Tris, pH 7.0, 5 mM beta-mercaptoethanol buffer resulted in a purified sample. The protein solution was not frozen but kept at 4 °C to avoid precipitation.

### Static secondary ion mass spectrometry (static SIMS)

Electrospray prepared targets were analyzed using time-of-flight secondary ion mass spectrometry (TOF-SIMS) I and II instruments^28–30^. The TOF-SIMS I instrument had a pulsed Ar^+^ ion source. The ions struck a grounded metal target. 8 keV Ar^+^ ions were focused to a 1 mm spot with a primary ion density of 10^10^ Ar^+^ ions per (cm^2^^2^ · second) (static SIMS conditions). Secondary ions were extracted from the grounded target and allowed to drift into two time-of-flight regions connected by a 163° angular and energy focusing toroidal Poschenrieder condenser lens. A single ion counter detector was used. Analytical ions were post-accelerated before being converted to electrons in a channel plate, which excited a scintillator. A photomultiplier counted the photon pulses. For the TOF SIMS II instrument, the Poschenrieder lens was replaced by an energy-compensating reflectron, which increased the mass resolution to more than 10 000. Improvements in the primary Ar^+^ ion source were important for this study. The ion current was more stable than in the TOF-SIMS I instrument, and the diameter of the ion beam on the target was 0.1 mm. This small focus was a significant improvement over older machines with an ion beam diameter of several millimeters and allowed for the first time the acquisition of spatially resolved scans of a target.

### Electron microscopy

Carbon-coated grids for electron microscopy were briefly exposed to gas discharge to render them hydrophilic. The on the liquid surface prepared layers were transferred to the electron microscopy grids by carefully lowering them onto the liquid meniscus. To extensively wash the specimens, they were exchanged several times with double distilled water. Then, the grids were negatively stained using 1% wt/vol uranyl acetate in preparation for electron microscopic inspection. Alternatively, the surface was covered with a 1.5 nm thick platinum/carbon layer through the use of an atomic beam angled at 15°. Inspection was conducted using an FEI Tecnai Spirit BioTWIN transmission electron microscope with varying magnifications of up to 150 000x, operating at 120 kV.

### Electrospray based surface preparation

#### Electrospray apparatus

An electrospray apparatus was constructed to uniformly dispense dissolved material onto a level surface^7^ (see Fig. 1 for the second generation instrument). The first generation instrument contained a metallic nozzle with a diameter of 250 μm. The flow rate was determined by the equilibrium between the electrostatic traction on the liquid and the flow resistance within the device. In the initial instrument the height of an accurately fitted metal cylinder in the outlet was modified to regulate its flow resistance. Like this, flow rates ranging from 8 to 15 µL/min were attainable. After the initial experiments, the nozzle was substituted with a hand-pulled glass capillary coated with gold, which lowered the stable flow rate to below 500 nanoliters/min^7^^7^. The liquid sample was sprayed under atmospheric conditions onto a flat metal target. By applying a high voltage, the liquid meniscus at the tip of the nozzle forms a Taylor Cone and emits a stream of highly charged, rapidly spreading small droplets from its tip^8^. The volatile solvent evaporates in flight, and the dried residues cover the target area in a circle approximately 4 mm in diameter. The distance between the nozzle and the target is adjustable.

For a second generation of the apparatus, the glass capillaries were made using a commercial capillary puller (model P-87 Puller, Sutter Instruments Company, Novato, CA, USA) and mounted in a pressurized container (Fig. 1). Prior to injection, the narrow glass tip was broken to provide an orifice approximately 1 μm in diameter^31^. With this more mature device, the electrospray operates at stable and constant flow rates between 20 and 100 nL/min.

#### Surface preparation

For the static secondary ion time-of-flight mass spectrometer (TOF-SIMS) study, a rhodamine/acetone solution was sprayed onto an aluminum target.

For the membrane preparation the solution was sprayed onto the liquid meniscus of a small 3 mm diameter cylindrical container filled with 10 mM Tris solution, pH 7.5, containing some SM-2 Biobeads (Bio-Rad, CA, USA). The container was connected to a larger buffer reservoir to compensate for evaporation losses. Like this the liquid meniscus was always high enough so that molecules, assembling a membrane on its surface, did not compete for space but could overflow.

Egg-phosphatidylcholine was purchased from Sigma-Aldrich.

The final membrane synthesis protocol for the incorporation of membrane proteins (like OmpG) consisted of five steps. The first material sprayed onto the buffer is the equivalent amount of a lipid bilayer of bipolar lipids dissolved in ethanol: 1 µL 66 picomoles/µL lipid with 35% cholesterol, 65% phosphatidylcholine. This preparation is incubated overnight at room temperature to allow for bilayer assembly. In a second step, a thin layer of glycerol is added by spraying a 1:2 solution of glycerol/ethanol onto the meniscus for about 10 min. The third step is to spray enough lipid to form a monolayer: 1 µL 33 picomoles/µL lipid with 35% cholesterol, 65% phosphatidylcholine in ethanol. In the fourth step, the sprayed membrane protein and lipid complete the molecular mixture to allow self-assembly of an intact, protein-containing lipid bilayer. The sprayed solution consisted of 25% glycerol to protect the protein from the high droplet charges when the water evaporates in flight: 1 µL 32 picomoles/µL OmpG, 20 picomoles/µL lipid 35% cholesterol, 65% phosphatidylcholine, 1% octylglucoside in 10 mM Tris, 25% glycerol. As a final step, another layer of glycerol is added to complete the hydrophilic environment: 1:2 glycerol/ethanol solution for approximately 10 min. The entire assembly is incubated for 6 days at room temperature to allow the SM-2 beads to extract the octylglucoside detergent from the surface layers.

The buffer solution used for the preparation of pneumolysin and listeriolysin O, proteins that are soluble in water by themselves but form a non-covalent intra-membraneous protein complex, contained 50 mM Tris and 150 mM NaCl. The pH value for listeriolysin O solution was adjusted to 5.5, for the pneumolysin solution to 7.0. The initial step in forming a protein membrane layer with initially soluble proteins involves spraying 1 µL of a 66 picomoles/µL lipid solution, containing 35% cholesterol and 65% phosphatidylcholine in ethanol, to allow for the formation of a closed lipid bilayer through overnight incubation. Spraying a 1:2 solution of glycerol/ethanol for about 10 min generates a thin layer of glycerol, creating a hydrophilic base for the subsequent assembly of the protein complexes. The following step involves the addition of either a lipid monolayer or a lipid bilayer. The bilayer was incubated overnight to complete its formation. At this point, the surface is ready for the addition of the protein-containing film. The pneumolysin solution had 26 picomoles/µL of protein in 50 mM Tris, 150 mM NaCl, at a neutral pH. The listeriolysin O solution had a concentration of 20 picomoles/µL, dissolved in 50 mM Tris, 150 mM NaCl, at pH 5.5. The solution that was sprayed had an additional component of 25% glycerol. When the surface consisted solely of a lipid monolayer, the protein solution provided enough lipid to complete the bilayer: 20 picomoles/µL lipid, with 35% cholesterol and 65% phosphatidylcholine. About 1 µl of the final solution distributed over the 3 mm liquid meniscus generates the protein-containing layer. Finalizing the hydrophilic environment involves spraying a 1:2 glycerol/ethanol solution for 10 min. The self-assembly process continues during the incubation period of several days at room temperature.

### Use of artificial intelligence

DeepL was used to bring the writing style of the manuscript closer to that of a native speaker (https://www.deepl.com/write).

## Electronic supplementary material

Below is the link to the electronic supplementary material.

Supplementary Material 1

Supplementary Material 2

## Data Availability

All relevant electron microscopic images are part of the article or its supplemental information. The overlap of the OmpG structure model derived from its crystal structure with the electron microscopic image has been made available via figshare (10.6084/m9.figshare.27074134) and the movie is part of the supplemental files.

## Abbreviations

OmpG: outer membrane protein G
E. coli: Escherichia coli
TOF-SIMS: Time of flight - secondary Ion mass spectrometry
