# Supplementary material for: Nanoelectrospray based synthesis of large, transportable membranes with integrated membrane proteins
Source: Sci Rep. 2024 Oct 24;14:25192. doi: 10.1038/s41598-024-76797-w (PMC11502708; doi:10.1038/s41598-024-76797-w)
Supplement: Supplementary file 2 — Supplementary Material 2 [file 41598_2024_76797_MOESM2_ESM.pdf]

Supplementary Information

Article Title:

Nanoelectrospray based synthesis of large, transportable membranes with integrated membrane proteins

Matthias Wilm*

Physics Institute of the University Münster, Surface Science, Münster, Germany

European Molecular Biology Laboratory (EMBL), Heidelberg, Germany

*Max Planck Institute for Biophysics, Frankfurt, Germany

Conway Institute, University College Dublin, Dublin, Ireland

Correspondence: Prof. Dr. Dr. Matthias Wilm, matthias.wilm@ucd.ie, Conway Institute, University College Dublin, Belfield, Dublin 4, Ireland

ORCID ID: 0000-0002-5461-6834

Supplementary Information

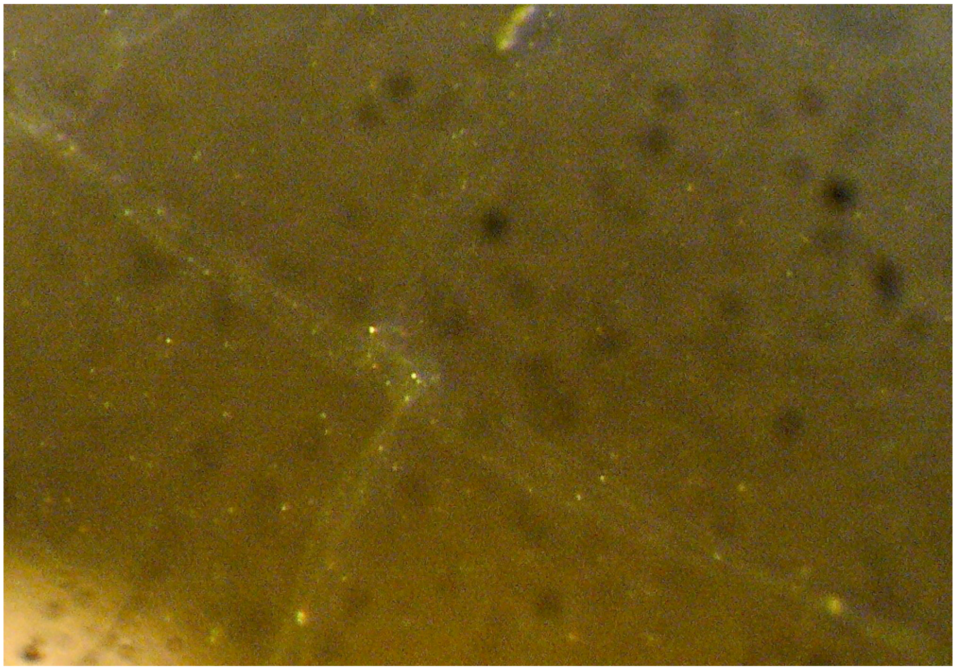

Supplementary Figure 1 Lipid Bilayer on Buffer

Stereomicroscopic image of a nanoelectrospray generated lipid bilayer on a buffer solution after overnight incubation at room temperature. Some folds of the viscous surface are visible.

Supplementary Video 1 Overlay of the Crystal Structure of OmpG and an OmpG Containing Membrane

Overlay of an OmpG crystal structure derived image (from Behlau, M., D. J. Mills, H. Quader, W. Kühlbrandt & J. Vonck. 2001. "Projection structure of the monomeric porin OmpG at 6 A resolution." *Journal of molecular biology* 305:71-77) with the platinum shadowed transmission electron image of the membrane containing OmpG (figure 4).

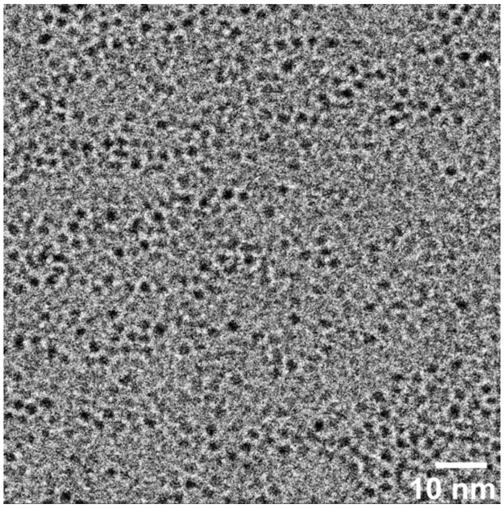

The scales of both images have been matched. The size and shape of the crystal structure derived image fits precisely to the structures seen in the electrospray prepared layers visualized in the transmission electron microscope. OmpG-Simulation.mp4 (figshare: https://doi.org/10.6084/m9.figshare.25908022)
